# Mutation in *Smek2* regulating hepatic glucose metabolism causes hypersarcosinemia and hyperhomocysteinemia in rats

**DOI:** 10.1038/s41598-022-26115-z

**Published:** 2023-02-21

**Authors:** Yasutake Tanaka, Michio Kawano, Sawako Nakashima, Chisato Yamaguchi, Makoto Asahina, Mai Sakamoto, Bungo Shirouchi, Kousuke Tashiro, Katsumi Imaizumi, Masao Sato

**Affiliations:** 1 Laboratory of Nutrition Chemistry, Division of Bioresource and Bioenvironmental Sciences, Graduate School, Kyushu University, Fukuoka, 819-0395 Japan; 2 Laboratory of Molecular Gene Technics, Department of Genetic Resources Technology, Faculty of Agriculture, Kyushu University, Fukuoka, 819-0395 Japan; 3 Laboratory of Nutrition Chemistry, Department of Bioscience and Biotechnology, Faculty of Agriculture, Graduate School, Kyushu University, 744 Motooka, Nishi-Ku, Fukuoka, 819-0395 Japan

**Keywords:** Mutation, Metabolic disorders, Liver, Liver diseases

## Abstract

*Suppressor of mek1 (Dictyostelium) homolog 2* (*Smek2*), was identified as one of the responsible genes for diet-induced hypercholesterolemia (DIHC) of exogenously hypercholesterolemic (ExHC) rats. A deletion mutation in *Smek2* leads to DIHC via impaired glycolysis in the livers of ExHC rats. The intracellular role of *Smek2* remains obscure. We used microarrays to investigate *Smek2* functions with ExHC and ExHC.BN-*Dihc2*^*BN*^ congenic rats that harbor a non-pathological *Smek2* allele from Brown-Norway rats on an ExHC background. Microarray analysis revealed that *Smek2* dysfunction leads to extremely low sarcosine dehydrogenase (*Sardh*) expression in the liver of ExHC rats. Sarcosine dehydrogenase demethylates sarcosine, a byproduct of homocysteine metabolism. The ExHC rats with dysfunctional *Sardh* developed hypersarcosinemia and homocysteinemia, a risk factor for atherosclerosis, with or without dietary cholesterol. The mRNA expression of *Bhmt*, a homocysteine metabolic enzyme and the hepatic content of betaine (trimethylglycine), a methyl donor for homocysteine methylation were low in ExHC rats. Results suggest that homocysteine metabolism rendered fragile by a shortage of betaine results in homocysteinemia, and that *Smek2* dysfunction causes abnormalities in sarcosine and homocysteine metabolism.

## Introduction

Exogenous hypercholesterolemic (ExHC) rats serve as a model of diet-induced hypercholesterolemia (DIHC)^1^. Although their serum cholesterol levels are within the normal range without dietary cholesterol, they rapidly develop hypercholesterolemia when fed with a diet containing cholesterol. We previously conducted linkage analysis of ExHC with Brown Norway (BN) rats as a control to identify the female-specific responsible loci Dihc1 on chromosome.5 and a unisex-responsible locus Dihc2, 4.1 Mbp region at the 3′ end, on chromosome.14^2,3^. Further investigation identified a responsible gene, suppressor of mek1 (Dictyostelium) homolog 2 (*Smek2*) from the Dihc2 region^3^. The ExHC rats have a 10-bp deletion mutation in the coding region of the *Smek2* gene^3^. This deletion leads to disrupted SMEK2 protein function that is not fatal in rats. With respect to the pathological mechanism of DIHC in ExHC rats, we found that compared with the original Sprague–Dawley (SD) strain, ExHC rats have impaired liver glucose metabolism (low expression of *liver-type phosphofructokinase, Pfkl*, a rate-limiting enzyme of glycolysis)^4^, low de novo fatty acid synthesis in the liver due to a shortage of required precursors^5^, and that this shortage of fatty acids leads to hepatic secretion of β-VLDL that is slowly uptaken by the liver^5^. However, the mechanism of *Pfkl* downregulation by the loss-of-function of *Smek2* is unclear. Furthermore, analyses of multiple changes in hepatic lipid metabolism might have overlooked some metabolic changes. The overall picture of *Smek2* function remains incomplete. Yoon et al.^6^ reported that SMEKs act as the activity-regulating domain of the transcriptional factor, cAMP response element-binding protein (CREB)-regulated transcription coactivator 2 (CRTC2). The ubiquitous transcriptional factor CREB regulates cell growth, differentiation, neuro-signals and other processes^7^. The CREB co-activators, CRTCs, regulate hormone secretion and nutritional metabolism. Abnormal transcriptional regulation can exert powerful effects in vivo, and gain- and loss-of-function mutations in transcriptional factors cause inherited anomalies and diseases^8–11^. For instance, mutations in hepatocyte nuclear factor-1a (HNF-1a) cause maturity-onset diabetes of the young (MODY3)^9^. Mutations in the CREB-binding protein (CREBBP) gene cause Rubinstein-Taybi syndrome (RSTS)^12^. Activated CREBBP acts on RNA polymerase via TFIIB, which is a basic transcription complex and promotes transcription^13^. An accumulation of abnormalities in the cascade reaction is thought to result in RSTS. Abnormal transcription factors are major problems. We previously found that a *Smek2* deficiency affects lipid metabolism^5^ and glucose metabolism^4^. Genes such as *Smek2*, which are not fatal if deficient, but can have widespread effects, are associated with the development of non-communicable diseases that manifest late in life. Here, we investigated the influence of dysfunctional *Smek2* in the livers of ExHC rats using microarray analysis.

Microarrays have been used for decades to exhaustively analyze gene expression^14,15^, and functions^16–18^, especially of target genes, such as LATS1 in human cell lines and PAP1 in Arabidopsis plants^17,18^. Microarrays are optimal for analyzing the influences of *Smek2* dysfunction. Here, we compared the effects of SMEK2 dysfunction between ExHC and congenic ExHC.BN*-Dihc2*^*BN*^ rats (congenic rats) that were established by backcross inbreeding during *Smek2* identification^3^, and harbor the low-responsive BN allele of Dihc2 on the high-responsive ExHC background genome. This comparison decreased noise derived from genetic differences between ExHC and congenic rats facilitated analyses of *Smek2* function. We also estimated the influence of the screened genes on metabolism in ExHC rats.

## Results

### Growth parameters and serum lipid levels

Table 1 shows the growth parameters and serum lipid levels. Initial and final body weight, as well as daily food intake did not significantly differ. However, the ExHC rats tended to gain weight (*P* = 0.053), and food efficiency was significantly increased. Dietary cholesterol did not affect growth parameters. Dietary cholesterol increased serum cholesterol levels in both strains, but significantly more so in ExHC, than in congenic rats. Serum triacylglycerol levels were significantly higher after 2% cholesterol diet in both strains. Serum phospholipid levels were significantly increased by dietary cholesterol and significantly higher in ExHC rats than congenic rats.

**Table 1:** Growth and biochemical parameters of ExHC and congenic (ExHC.BN-*Dihc2*^*BN*^) rats.

	Group				P		
	Cholesterol (−)		Cholesterol (+)		Strain	Chol	S × C
	Congenic	ExHC	Congenic	ExHC			
Initial body weight (g)	92.1 ± 1.7	92.5 ± 1.7	92.1 ± 1.3	89.3 ± 2.2	NS	NS	NS
Final body weight (g)	175 ± 3	175 ± 3	177 ± 2	169 ± 3	NS	NS	NS
Body weight gain (g)	83.5 ± 1.9	82.3 ± 1.7	84.9 ± 1.5	79.5 ± 1.5	0.053	NS	NS
Dietary food intake (g/day)	15.3 ± 0.2	15.4 ± 0.2	15.4 ± 0.2	15.7 ± 0.1	NS	NS	NS
Food efficiency (g body weight gain/g food intake)	0.454 ± 0.007	0.446 ± 0.004	0.452 ± 0.009	0.43 ± 0.01	< 0.05	NS	NS
Liver weight (g)	10.2 ± 0.3	9.8 ± 0.2	11.4 ± 0.19	10.2 ± 0.3	< 0.01	< 0.01	NS
**Serum parameters**							
Cholesterol (mg/dL)	82.1 ± 1.6^a^	91.4 ± 1^a^	206.3 ± 9.7^b^	432.9 ± 44.8^c^	< 0.01	< 0.01	< 0.01
Free cholesterol (mg/dL)	26.9 ± 0.6^a^	27.9 ± 1.2^a^	35 ± 1.7^a^	88.5 ± 17^b^	< 0.01	< 0.01	< 0.01
Esterified cholesterol (mg/dL)	55.2 ± 1.2^a^	63.5 ± 1.1^a^	171.3 ± 8.3^b^	344.4 ± 30.4^c^	< 0.01	< 0.01	< 0.01
HDL-cholesterol (mg/dL)	45.2 ± 1.6	49.7 ± 1.6	33.5 ± 2.9	31.6 ± 2.6	NS	< 0.01	NS
NonHDL-cholesterol (mg/dL)	37 ± 0.9^a^	41.8 ± 2.1^a^	172.8 ± 10.6^b^	401 ± 46^c^	< 0.01	< 0.01	< 0.01
Triacylglycerol (mg/dL)	242 ± 15	205 ± 18	140 ± 17	132 ± 17	NS	< 0.01	NS
Phosoholipids (mg/dL)	207 ± 4	224 ± 11	228 ± 8	274 ± 18	< 0.01	< 0.01	NS
Glucose (mg/dL)	160 ± 5^ab^	171 ± 4^a^	153 ± 2^b^	199 ± 5^c^	< 0.05	< 0.01	< 0.01
**Liver parameters**							
Cholesterol (mg/g liver)	5.08 ± 0.35	36.9 ± 1.83	5.05 ± 0.28	36.4 ± 0.85	NS	< 0.01	NS
Free cholesterol (mg/g liver)	1.9 ± 0.16	5.85 ± 0.53	1.64 ± 0.05	5.34 ± 0.65	NS	< 0.01	NS
Esterified cholesterol (mg/g liver)	3.18 ± 0.48	31 ± 1.8	3.42 ± 0.31	30.7 ± 0.9	NS	< 0.01	NS
Total PL (mg/g liver)	20.3 ± 0.3	20.1 ± 0.8	20.3 ± 0.2	19.2 ± 1	NS	NS	NS
PC (mg/g liver)	11.4 ± 0.2	11.6 ± 0.5	11.3 ± 0.1	11 ± 0.6	NS	NS	NS
PE (mg/g liver)	6.68 ± 0.09	6.08 ± 0.27	6.74 ± 0.09	5.68 ± 0.31	< 0.01	NS	NS
PS + PI (mg/g liver)	2.05 ± 0.06	2.13 ± 0.1	1.85 ± 0.06	2.05 ± 0.21	NS	NS	NS
SPH (mg/g liver)	0.193 ± 0.018	0.287 ± 0.085	0.42 ± 0.04	0.504 ± 0.079	NS	< 0.01	NS
PC/PE	1.71 ± 0.02	1.92 ± 0.04	1.68 ± 0.03	1.93 ± 0.03	< 0.01	NS	NS

Values are shown as means ± SEM. n = 6/group, Values of total PL represent the sums of PC, PE, PS + PI, and SPH. Levels of esterified and non-HDL-cholesterol were calculated as differences between total and free cholesterol level and between total cholesterol and HDL-cholesterol, respectively. Statistical differences were assessed using two-way ANOVA followed by Tukey–Kramer post-hoc tests. ^a,b,c^Significant differences between groups with different letters at *P* < 0.05.

### Microarray findings

**Figure 1:**
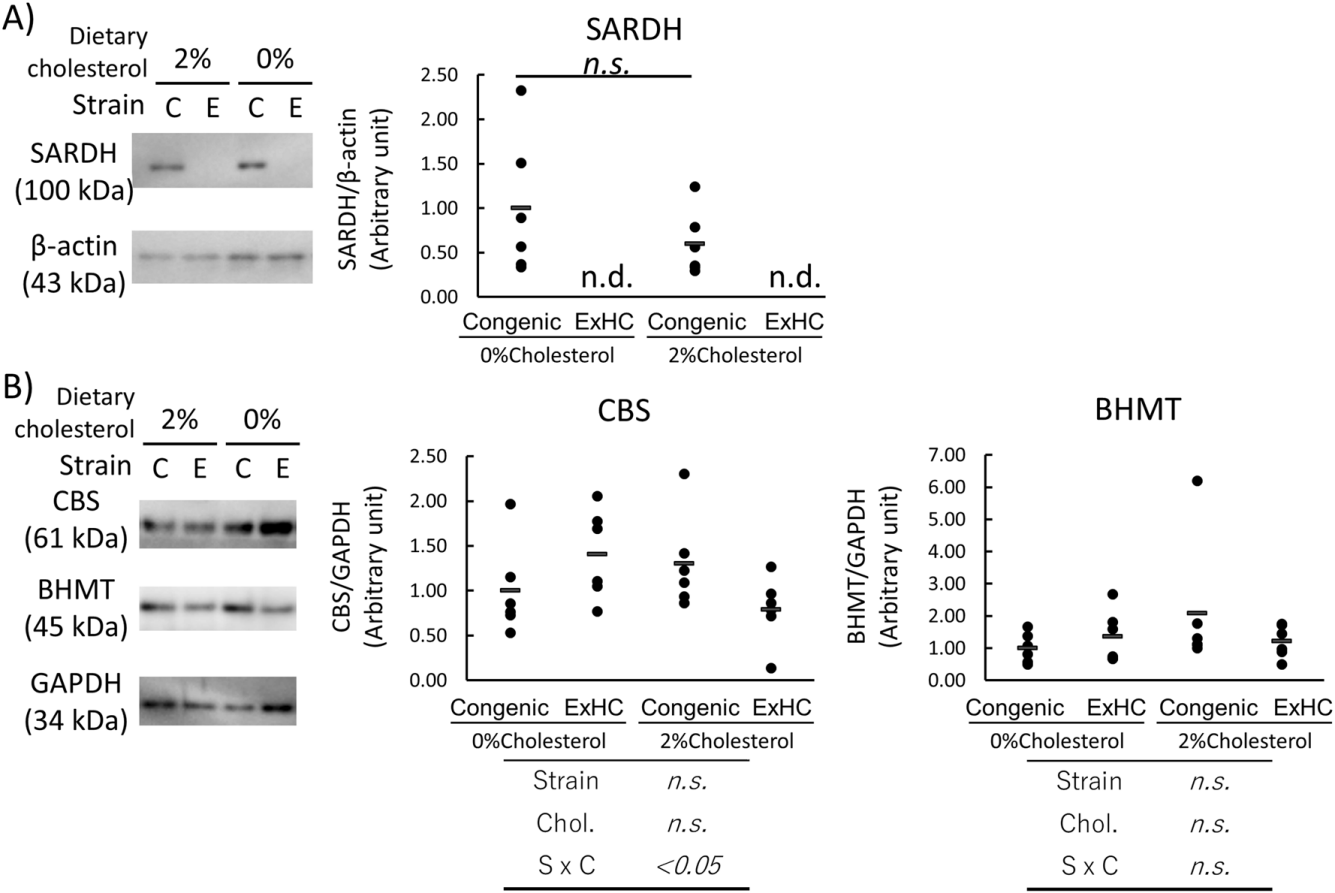
Protein expressions in the livers of congenic (ExHC.BN-*Dihc2*^*BN*^) (C) and ExHC (E) rats. (**A**) SARDH, (**B**) CBS and BHMT. n = 6/group, Dots and bar in each graph represent the values of each rats and the average of group, respectively. Differences among groups were analyzed with two-way ANOVA with Tukey–Kramer post-hoc test. n.d.; not detected, n.s.; not significant.

Microarray analysis revealed 251 and 377 genes that were upregulated and downregulated, respectively, in ExHC, compared with congenic rats (data not shown). Table 2 shows the top 10 genes with downregulated expressioned in ExHC rats, the most significant being the mRNA expression of *Sardh*, encoding sarcosine dehydrogenase. The binary logarithm of fold change of expression levels in ExHC rats to congenic rats was − 9.571 (~ 1/760 of congenic rats). Protein expressions of SARDH was not observed in the liver of ExHC rats regardless of dietary cholesterol contents (Fig. 1).

**Table 2:** Genes affected by dysfunctional *Smek2* in ExHC rats.

Rank	Symbol	Gene name	P	Log
1	Sardh	Rattus norvegicus sarcosine dehydrogenase (Sardh), mRNA [NM_053664]	1.56.E−06	− 9.571
2	RT1-DO6	Rattus norvegicus RT1 classII, locus Dob (RT1-DO6), mRNA [NM_001008846]	4.42.E−03	− 5.765
3	CO567719	AGENCOURT_28449398 NIH_MGC_248 rattus norvegcus cDNA clone IMAGE: 73656285 mRNA sequence [CO567719]	8.65.E−04	− 5.531
4	Ar	Rattus norvegicus androgen receptor (Ar), mRNA [NM_012502]	2.42.E−03	− 5.528
5	AI013540	AI013540 ES T208215 Normalized rat spleen, Bento Soares Rattus sp. cDNA clone RSPB003 3′ end mRNA sequence [AI013540]	9.56.E−03	− 5.252
6	Ar	Rattus norvegicus androgen receptor (Ar), mRNA [NM_012502]	4.82.E−03	− 5.228
7	BI274340	BI274340 UI-R-CW0-bwh-g-06-UI.s1 UI-R-CW0 rattus norvegicus cDNA clone UI-R-CW0-bwh-g-06-0-UI 3′, mRNA sequence [BI274340]	7.29.E−03	− 5.145
8	Zfp647_predicted	Rattus norvegicus similar to Hypothetical protein 6030449J21 (LOC362948), mRNA [XM_343279]	5.02.E−03	− 5.060
9	BQ209739	BQ209739 UI-R-DZ1-cog-e-05-0-UI.s1 NCI_CGAP_DZ1 Rattus norvegicus cDNA clone IMAGE:7348303 3′, mRNA sequence [BQ209739]	9.58.E−03	− 4.838
10	M23948	RATCARB02 Rat carboxypeptidase B gene, intron 1, middle region A [M23948]	6.48.E−03	4.469

### Determination of hepatic mRNA expression

Table 3 shows the hepatic mRNA expression of genes associated with homocysteine metabolism. The mRNA expression of *Sardh* and betaine-homocysteine methyltransferase (*Bhmt*) was significantly lower, and that of cystathionine beta-synthase (*Cbs*) and of phosphatidylethanolamine N-methyltransferase (*Pemt*) was significantly higher in ExHC than in congenic rats. In contrast, dietary cholesterol intake significantly decreased *Bhmt* and *Pemt* mRNA levels in both rat strains. Although an interaction was found between strain and dietary cholesterol in *Smek2*, there was no significant difference by dietary cholesterol with Tukey–Kramer post-hoc test.

**Table 3:** Hepatic mRNA expression in ExHC and congenic rats.

Symbol	Group				P		
	Cholesterol (−)		Cholesterol (+)		Strain	Chol	S x C
	Congenic	ExHC	Congenic	ExHC			
*Sardh*	1.00 ± 0.05	0.00 ± 0.00	1.12 ± 0.06	0.00 ± 0.00	< 0.01	NS	NS
*Dmgdh*	1.00 ± 0.04	1.04 ± 0.02	1.00 ± 0.06	1.00 ± 0.05	NS	NS	NS
*Bhmt*	1.00 ± 0.12	0.78 ± 0.05	0.80 ± 0.06	0.57 ± 0.06	< 0.01	< 0.05	NS
*Cbs*	1.00 ± 0.05	1.16 ± 0.03	0.99 ± 0.09	1.15 ± 0.08	< 0.05	NS	NS
*Gnmt*	1.00 ± 0.04	1.01 ± 0.03	1.00 ± 0.04	0.99 ± 0.07	NS	NS	NS
*Gamt*	1.00 ± 0.03	1.08 ± 0.06	1.02 ± 0.09	0.96 ± 0.04	NS	NS	NS
*Pemt*	1.00 ± 0.02	1.09 ± 0.05	0.85 ± 0.03	0.92 ± 0.09	NS	< 0.01	NS
*Pcyt1a*	1.00 ± 0.06	0.97 ± 0.08	0.92 ± 0.12	0.98 ± 0.09	NS	NS	NS
*Mthfr*	1.00 ± 0.16	1.32 ± 0.16	0.85 ± 0.14	1.29 ± 0.19	< 0.05	NS	NS
*Smek2*	1.00 ± 0.07^a^	0.21 ± 0.02^b^	1.32 ± 0.14^a^	0.19 ± 0.03^b^	< 0.01	NS	< 0.01

Values are shown as means ± SEM. n = 6/group, Mean value of cholesterol (−) -congenic rats was set to 1.00. The Gusb gene was as the internal standard. Differences for each gene were detected by two-way ANOVA followed by Tukey–Kramer post-hoc test and were considered significant at *P* < 0.05. NS in the table is the abbreviation for “not significant”. ^a,b,c^Significant differences between groups with different letters at *P* < 0.05.

### Determination of hepatic protein expression

Figure 1 shows the hepatic protein expressions of SARDH, CBS, BHMT in the liver. In the liver of ExHC rats, SARDH protein was not detected (Fig. 1A). There was no significant difference on the expressions of BHMT (Fig. 1B). Although the interaction between strain and dietary cholesterol on CBS expression was significant, there was no significant difference after Tukey–Kramer post-hoc test.

### Analysis of serum and liver parameters

Table 4 shows the serum and hepatic parameters. Serum sarcosine and homocysteine levels were significantly increased in ExHC rats regardless of the presence or absence of dietary cholesterol, and serum betaine levels were decreased compared with those of congenic rats. Levels of hepatic sarcosine were significantly higher, and those of PE were lower (increased PC/PE ratio) in ExHC compared with congenic rats. Although dietary cholesterol did not affect serum sarcosine levels, dietary cholesterol decreased hepatic sarcosine and increased hepatic SPH levels, regardless of strain. No interaction was found between strain and dietary cholesterol in any of the parameters.

**Table 4:** Serum and hepatic parameters associated with sarcosine metabolism in ExHC and congenic rats.

	Group				*P*		
	Cholesterol (−)		Cholesterol (+)		Strain	Chol	S x C
	Congenic	ExHC	Congenic	ExHC			
**Serum parameters**							
Sarcosine (μM)	52.2 ± 2.4	300 ± 17	41.4 ± 2.4	300 ± 12	< 0.01	NS	NS
Betaine (μM)	74.9 ± 2.4	55.0 ± 1.8	76.6 ± 1.3	53.2 ± 0.8	< 0.01	NS	NS
Homocysteine (μM)	15.0 ± 1.1	25.3 ± 2.8	16.7 ± 1.6	34.2 ± 2.3	< 0.01	NS	NS
**Liver parameter**							
Sarcosine (μg/g liver)	4.81 ± 0.45	11.4 ± 0.5	4.09 ± 0.60	10.4 ± 0.4	< 0.01	< 0.05	NS

Values are shown as means ± SEM. n = 6/group, S × C, strain × cholesterol interaction. *P, two-way ANOVA. NS in the table is the abbreviation for “not significant”.

### Determination of *Sardh* mRNA levels in cells with *Smek2* knockdown

**Figure 2:**
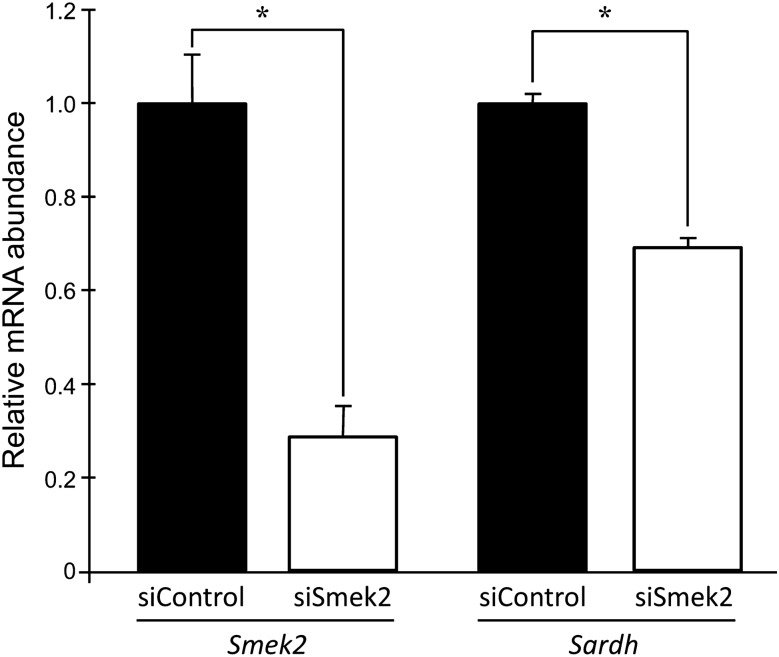
Gene expression in MCA-RH 7777 rat hepatoma cells with *Smek2* siRNA. Values are shown as means ± SEM. n = 4/group, *Significantly different at *P* < 0.01 by Student’s *t*-test.

Small-interfering RNA (siRNA) against Smek2 suppressed *Smek2* mRNA expression by 80% and *Sardh* mRNA expression by 31% in rat hepatocytes (Fig. 2).

## Discussion

**Figure 3:**
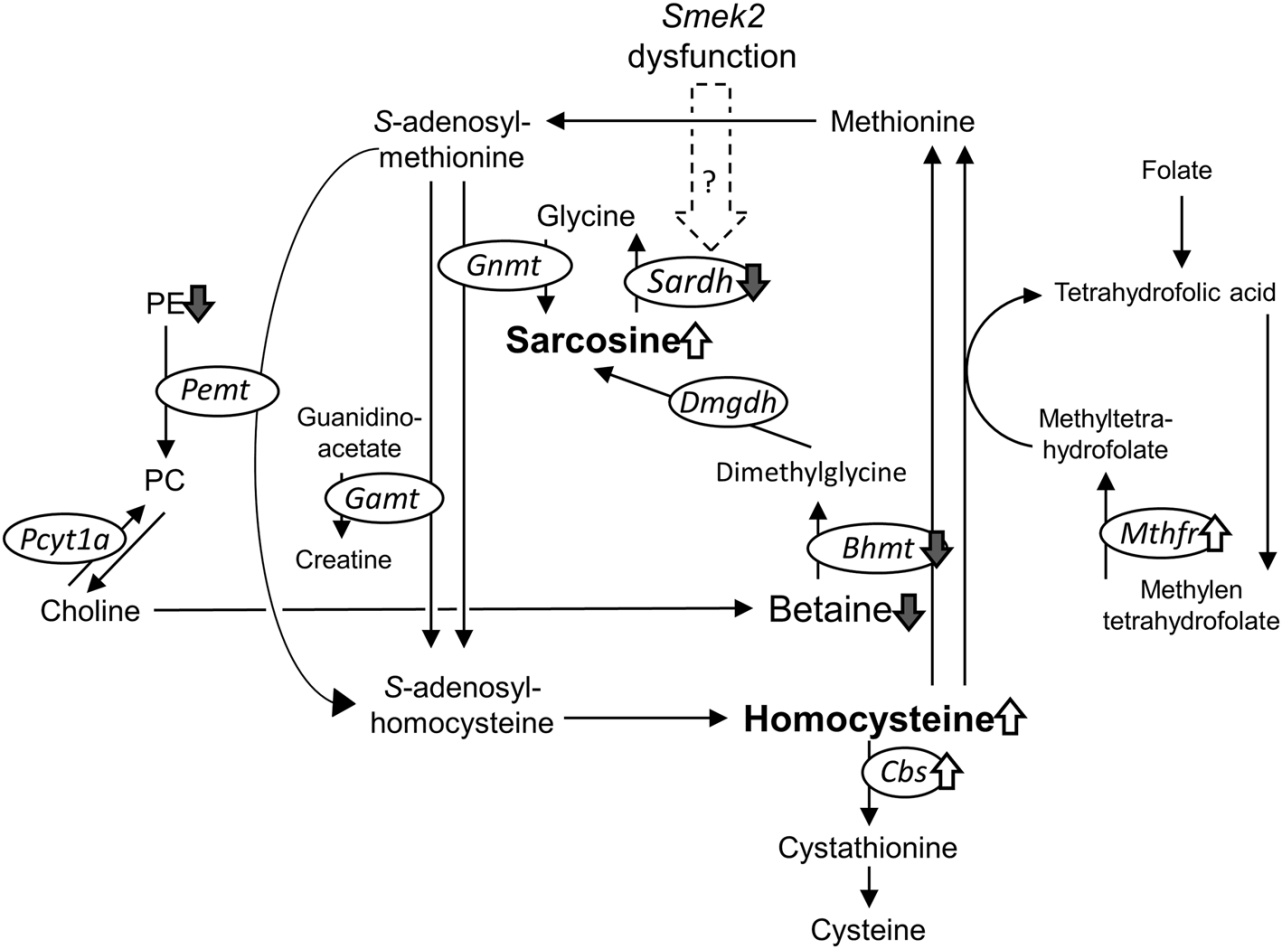
Diagram of methionine/homocysteine metabolism. Reactions are shown as arrows in lines. Gene symbols for related enzymes are described in circles. *Bhmt*; betaine-homocysteine methyltransferase, *Cbs*; cystathionine beta-synthase, *Dmgdh*; dimethylglycine dehydrogenase, *Gamt*; guanidinoacetate methyltransferase, *Gnmt*; glycine N-methyltransferase, *Ms*; methionine synthase, *Mthfr*; methylenetetrahydrofolate reductase, *Pcyt1a*; phosphate cytidylyltransferase 1, *Pemt*; phosphatidylethanolamine N-methyltransferase, *Sardh*; sarcosine dehydrogenase.

We investigated the function of the *Smek2* gene, which is responsible for DIHC^3^, by comparing the transcriptomes of ExHC (tentatively *Smek2*^−/−^) and congenic (tentatively *Smek2*^+/+^) rats. The pathogenic ExHC allele of *Smek2* exacerbated the response to dietary cholesterol (approx. + 223% to congenic rats) and increased serum phospholipid levels (+ 8 ~ 20%). Dietary cholesterol decreased serum triacylglycerol levels by approx. 40% in both strains. These observed changes were consistent with the previously reported results^3–5,45^. The findings revealed that ExHC rats developed hypersarcosinemia due to *Smek2* dysfunction via dysregulated *Sardh* mRNA expression. Sarcosine (monomethylglycine) is synthesized from glycine by *S*-adenosylmethionine demethylation (Fig. 3). Sarcosine accumulation is related to emotional instability, growth failure^19^, and prostate cancer^20^ in humans. However, the relationship between sarcosine metabolism and the onset of these diseases has remained obscure. A *sar*^*−/−*^ mouse model of hypersarcosinemia with a mutation in *Sardh* has been established, and this mouse develops average plasma sarcosine concentrations of 785 μM^21^. However, the sarcosine content of individual tissues from *sar*^*−/−*^ mice has not been investigated. Humans with *SARDH* mutations also develop hypersarcosinemia^22^ and have rather variable plasma sarcosine levels (760–53 μM)^19^. The normal range of plasma sarcosine is 3–0.5 μM in healthy persons^19^. However, sarcosine metabolism is regulated by the dietary intake of nutrients associated with one-carbon metabolism, such as folic acid and cobalamin^23,24^. Hypersarcosinemia is induced in humans who are folic acid-deficient, with sarcosine levels ranging from 40 to 0.6 μM^25^. The ExHC rats (average sarcosine level: 300 μM) were hypersarcosinemic based on these values. During homocysteine metabolism, sarcosine is formed by glycine methylation (Fig. 3). A folate deficiency typically induces homocysteinemia^26^, and the ExHC rats developed both hypersarcosinemia and homocysteinemia^21^. ExHC rats developed abnormal amino acid levels in blood in addition to abnormal lipid parameters observed previously^5^. This is the first study to show that abnormal sarcosine metabolism caused by decreased *Sardh* expression in the liver can lead to homocysteinemia. The blood homocysteine levels normal SD rats were approximately 5 μM^27^ and increased to approximately 20 μM due to deficiency of vitamin B12 or folic acid^27^. Mutations in *Bhmt*, betaine-homocysteine methyltransferase, induce serum homocysteine levels to ~ 50 μM in mice^28^. Homocysteinemia was relatively severe (average 30 μM) in ExHC, compared with congenic rats that developed mild homocysteinemia (average 15 μM). Taken together, these results indicated that the ExHC rats had developed hypersarcosinemia with homocysteinemia.

Serum homocysteine levels in ExHC rats were approximately 50% of those in congenic rats. The 4.1 Mbp region on chromosome.14 of the congenic rats was derived from Brown Norway (BN) rats, and the rest of their genome was derived from ExHC rats. These facts indicate that an important regulator of *Sardh* is located in the Dihc2 region, which contains 33 genes including *Smek2*^3^*.* However, the change was insufficient to explain why a similar change to that of *Sardh* expression was not evident in any other gene except *Smek2* between ExHC and congenic rats^3^. Since *Smek2* knockdown reduced *Sardh* expression in rat hepatocytes (Fig. 2), *Smek2* is probably involved in *Sardh* regulation. The Smek2 protein can regulate protein phosphatase 4 (PP4)^6^, which is required for expression of the stress response transcriptional regulator, DAF-16^29^, which in turn is required for high-homocysteine-stress resistance in the nematode *C. elegans*^30^. Another study has shown that Smek suppresses the expression of Wnt signaling genes by promoting histone deacetylase^31^. Activation of the Wnt/β-catenin signal suppresses sterol regulatory element binding protein-1c (SREBP-1c), the master transcriptional regulator of lipid synthesis^32,33^. The expression of SREBP-1c is not affected in the ExHC rat liver^5^. Therefore, Wnt signaling might contribute little to *Smek2* function. Sarcosinemia and homocysteinemia in ExHC rats might be mediated by these or other pathways rather than being directly regulated by *Smek2*.

Homocysteinemia is induced experimental animals by a deficiency of dietary vitamin B12 or folic acid^23,24^. Both cause a depression in homocysteine catabolism (methionine cycle) due to the depression of the folic acid metabolism. The nutritional conditions between ExHC and congenic rats did not significantly differ in the present study, so these deficiencies did not cause homocysteinemia. Betaine is a methyl donor for the conversion of homocysteine to methionine. Lower serum betaine levels suggested that homocysteinemia in ExHC rats is caused by a delay in homocysteine catabolism due to a betaine deficiency. Betaine is normally generated by the conversion of choline or glycine via sarcosine. The excessive accumulation of sarcosine in hepatocytes implied that betaine synthesis from glycine was low in ExHC rats. Additionally, a decrease in PE indicated that betaine synthesis from choline was also low in ExHC rats. This was considered to be a result of preferentially synthesizing PC, which is necessary for the formation of cell and lipoprotein membranes, from choline as well as betaine. The betaine deficiency in the livers of ExHC rats was caused by a lack of hepatic choline. Phosphatidylcholine is needed to construct stable lipid membranes because it has less lipid bilayer flexion than PE, PA, and lysophospholipids^34,35^. The ExHC rat liver slowly re-uptakes β-VLDL secreted from it^5^. The hepatic PC requirement in ExHC rats might be higher than that of other rat strains. Additionally, a decrease in PC supply from the de novo synthesis pathway, which is another source of PC, might be suppressed in ExHC rats because of the impaired fatty acid synthesis. In fact, PE, which is also a precursor of PC, was reduced in the livers of ExHC rats. Taken together, betaine was reduced because choline was preferentially used for PC synthesis in the liver of ExHC rats. As another regulatory factor, genes involved in one-carbon metabolism such as *Bhmt* and *Sardh* are regulated by intracellular and extracellular osmotic pressure^36^. Osmotic pressure around hepatic cells might be high because large amounts of sarcosine solute accumulated in the livers of ExHC rats. Hepatic *Bhmt* mRNA levels were significantly corelated with hepatic sarcosine levels (R = − 0.59, *P* < 0.01) and serum homocysteine levels (R = − 0.55, *P* < 0.01). However, hepatic BHMT protein expression levels show no significant co-relationship. Similar results were observed about hepatic CBS expressions. There should be a proportional relationship between mRNA levels and protein levels, but CBS and BHMT in this study did not. Since reports on post-translational regulation of CBS and BHMT were not found, the detail of this unlink between mRNA and protein levels was unknown. As one possibility, hepatic protein levels of BHMT and CBS may be saturated both in congenic and ExHC rats since both strains also develop homocysteinemia as described above. In this study, because the amount of increases in *Cbs* mRNA levels was as small as 15%, and the protein levels were not linked, CBS was considered to be unrelated to development of homocysteinemia. On the other hand, *Bhmt* mRNA levels decreased by *Smek2* dyfunction, and by cholesterol feeding, respectively. But, BHMT protein levels were not changed in this study. There is a report that BHMT protein was also proportionally decreased linked to mRNA levels in high-fat-diet fed mouse^37^. As another possibility that BHMT protein levels did not reflect mRNA levels, *Smek2* dysfunction may affect BHMT protein regulation in the liver. In any case, the changes of enzymes related to homocysteine metabolism were not direct causes for severe homocysteinemia in ExHC rats. Taken together, hepatic choline metabolism might be involved in the regulation of homocysteine metabolism in ExHC rats.

The decreased PC/PE ratio of the liver is linked to the progression of fatty liver and NASH in rats^38,39^. The normal range of the hepatic PC/PE ratio in mice is 1.5–2.0^40^. The PC:PE ratios of both congenic and ExHC rats in this study were within this range. The PC:PE ratio of 1.9 indicated that the liver remained normal in ExHC rats. The present study showed that anomalies in *Smek2* resulted in disrupted sarcosine, homocysteine, lipid, and carbohydrate metabolism, but might be protective only in terms of maintaining a robust hepatic lipid composition.

In conclusion, microarray analysis revealed that hepatic mRNA levels of *Sardh* (sarcosine dehydrogenase) were significantly lower in ExHC (*Smek2*^−/−^), than in congenic (*Smek2*^+/+^) rats. The ExHC rats developed hypersarcosinemia and further homocysteinemia due to abnormal sarcosine metabolism caused by decreased *Sardh* expression. In addition to regulating glucose metabolism^4,5^, *Smek2* is also important for regulating amino acid metabolism.

## Materials and methods

### Animals and diets

Ethical approval for the experiments was granted by the Animal Care and Use Committee, Kyushu University (Authorization no.: A22-160-2). This study proceeded according to the guidelines for animal experiments at the Faculty of Agriculture and Graduate Course at Kyushu University. The study was carried out in compliance with the ARRIVE guidelines. Male ExHC/Sea and congenic rat colonies were maintained through brother-sister mating (Nutrition Chemistry Laboratory, Faculty of Agriculture, Kyushu University). All rats had free access to an NMF, non-purified diet (Oriental Yeast Co., Tokyo, Japan) and deionized water in a temperature-controlled room at 22–25 °C with a 12-h light cycle (0800–2000). Control and cholesterol diets were formulated according to the AIN76™ formula^41^ as (weight %): 0 or 2 cholesterol, 10 olive oil (fatty acid composition %: 10.1 palmitic acid, 0.6 palmitoleic acid, 3.8 stearic acid, 80.7 oleic acid, 4.1 linoleic acid, 0.4 α-linolenic acid, 0.3 eicosenoic acid, fatty acid as described^5^, 20 casein, 15 corn starch, 5 cellulose, 3.5 mineral mixture (AIN76™), 1 vitamin mixture (AIN76™), 0.3 _DL_-methionine, 0.2 choline bitartrate and sucrose to 100. Rats of each strain were randomly assigned to control diet group or cholesterol diet group (n = 6/group) at 4 weeks old. In this study, we chose a small sample size to screen for genetic alterations that were not found in previous studies. Therefore, the original intention was to collect basic evidence of genetic variation in a simple design of experiments. After feeding with these diets for 2 weeks, the rats were beheaded and blood samples were collected. Livers were immediately excised and serum was separated from blood by centrifugation at 1750×*g* at 4 °C for 5 min.

### Determination of serum lipid levels

Serum cholesterol, triacylglycerol and phospholipid levels were measured using enzyme kits (T-CHO KAINOS, Triglyceride E-test WAKO and Phospholipid C-test WAKO) (KAINOS Laboratories, Inc., Tokyo, Japan and Fujifilm Wako Pure Chemical Corporation, Osaka, Japan).

### RNA extraction

Total cellular RNA was isolated from liver tissues and cultured cells using the acid-guanidium-phenol-chloroform (AGPC) method as described^5^ and incubated with DNase I (Thermo Fisher Scientific Inc., Waltham, MA, USA) at 37 °C for 30 min to prevent DNA contamination.

### Microarray analysis

#### Synthesis of *Cy3-labeled* cRNA probe

Samples of RNA from rats fed with cholesterol (n = 4/group) were analyzed using microarrays. Since the number of individuals that can be analyzed by microarray at one time was limited, four rats per group were selected with serum cholesterol levels close to the averages of each group. Cyanine3 (Cy3)-labeled complementary RNA (cRNA) was synthesized with following kits as described by the manufacturers. Complementary DNA (cDNA) was synthesized from RNA samples selected using RNA Spike-In Kit, One-Color (Agilent Technologies Inc., Santa Clara, CA, USA). Cy3-labeled cRNA was synthesized from cDNA, amplified using Quick Amp Labeling Kit, One-Color (Agilent), then purified using RNeasy mini spin columns (Qiagen, Hilden, Germany).

#### Microarray hybridization and data analysis

Cy3-labeled cRNA (1.65 μg) was fragmented using Covaris M (Covaris Inc., Woburn, MA, USA), then hybridized to the Whole Rat Genome (4 × 44 K) Oligo Microarray (Agilent) using a Gene Expression Hybridization Kit (Agilent). Slides were washed three times with Gene Expression Wash Buffers 1 and 2 (Agilent). The slides were dried and visualized using an Agilent Microarray Scanner (Agilent). The density of spots was measured by Feature Extraction, the software provided with the microarray scanner. After background correction and normalization, we compared levels of gene expression corresponding to the density of each spot, between the ExHC and congenic rats.

### Determination of hepatic protein levels

Hepatic protein levels of SARDH (100 kDa), CBS (61 kDa), and BHMT (45 kDa), were measured by western blotting. Liver tissues (50 mg) were homogenized in 500 μL of 10 mM Tris–HCl containing 1 mM EDTA (pH 7.4) using a biomasher. Protein concentrations in liver homogenates were measured with a commercial kit (DC™ Protein Assay) (Bio-Rad Laboratories, Inc., CA, USA). The lysate solutions containing 50 ug protein were transferred to an 0.6 ml-tubes, vacuum dried, and then redissolved in 50 µl of sample buffer (0.M Tris–HCl, 2% SDS, 10% glycerol, 1.55% dithiothreitol). This protein solutions (15 µl/well) were applied to SDS-PAGE (5% acrylamide gel for stacking, 10% acrylamide gel for separating). Protein in gel was transblotted on Amersham™ Hybond™ P0.45 PVDF membrane (GE Healthcare, IL, USA). The blocking of the PVDF membrane was done with blocking reagent (Blocking One, Nacalai tesque, Kyoto, Japan). Each protein on the membrane was stained with anti-SARDH rabbit IgG antibody (1:3000) (22762-1-AP) (Proteintech Group, Inc., IL, USA), anti-CBS rabbit IgG antibody (1:3000) (14787-1-AP) (Proteintech), and anti-BHMT rabbit IgG antibody (1:3000) (15965-1-AP) (Proteintech) for 1st antibody, and anti-rabbit IgG antibody (1:3000) (NA934) (Merck KGaA., Darmstadt, Germany) for 2nd antibody. Antibodies were with Tween20-containing phosphate buffered saline (PBS-T) containing 1% Skim Milk for immunoassay (Nacalai tesque). As an internal standard protein, hepatic levels of β-actin (43 kDa) or GAPDH (34 kDa) were detected with anti-β-actin rabbit IgG antibody (1:3000) (Code: PM053) (Medical & biological laboratories Co., LTD., Tokyo, Japan) anti-GAPDH mouse IgG antibody (1:25,000) (60004-1-AP) (Proteintech), anti-rabbit IgG antibody and anti-mouse IgG antibody (1:3000) (NA931) (Merck KGaA). BHMT expression was measured next to GAPDH. For re-probing of BHMT, anti-GAPDH IgG antibody on the membrane was stript with WB Stripping Solution (Code# 05680-21) (Nacalai tesque). These proteins were detected with ECL™ Prime Western Blotting Detection Reagent (GE Healthcare) and LAS4000 (GE Healthcare). Intensities of observed protein bands were measured with KyPlot 6.0 (KyensLab Inc.) (https://www.kyenslab.com/en-us/). Whole  pictures  of  menbranes were  provided as Supplemental figures (S1, S2).

### *Smek2* knockdown

#### Preparation of lipoprotein deficient serum (LPDS)

We prepared lipoprotein deficient serum (LPDS) from Gibco (LOT#366208) fetal bovine serum (FBS) (Thermo Fisher Scientific Inc.). A solution of potassium bromide (32.8 g) in 100 mL of FBS was ultracentrifuged at ~ 145,000×*g* using an RP-55T rotor (Hitachi Koki Co., Tokyo, Japan) for 40 h in an SCP70H2 ultracentrifuge (Hitachi Koki Co.), then LPDS was collected from the ultracentrifuge tubes and dialyzed against PBS twice for 1 h each, then overnight at 4 °C. Dialyzed LPDS sterilized using syringe filters with 0.45-μm pores (Whatman International Ltd., Maidstone, UK) was included in cell culture medium.

#### Cell culture

MCA-RH7777 rat hepatoma cells (DS Pharma Biomedical Co., Ltd., Osaka, Japan) were maintained in basal media, DMEM (pH 6.9) (Thermo Fisher Inc.) containing 10% v/v FBS, 1.0 × 10^5^ IU/L penicillin, 0.1 g/L streptomycin, and 3.7 g/L NaHCO_3_ under a 5% CO_2_ atmosphere at 37 °C. Cells that reached 80%–90% confluence were subcultured for 48 h, then diluted to 3 × 10^5^/mL in NEAA (non-essential amino acid) medium, DMEM containing 10% v/v LPDS, 0.28 mM alanine, 0.33 mM asparagine, 0.35 mM proline monohydrate, 0.23 mM asparagusic acid, 0.51 mM glutamic acid.

#### *Smek2* siRNA treatment

Diluted cells (1 mL) were seeded in 12-well plates and incubated for 24 h. Thereafter, 200 μL of siRNA-lipid complex, DMEM containing 0.075 μM *Smek2* siRNA or negative control siRNA (Cosmobio Co., Ltd., Tokyo, Japan) (Table 5), and Lipofectamine RNAi MAX (1.5 μL; Thermo Fisher Scientific Inc.) were added to the wells. The medium was replaced 24 h after transfection, then total RNA was extracted from the cells after 24 h thereafter.

**Table 5:** Oligonucleotides for *Smek2* siRNA.

		Sequence
*Smek2* R 128	Sense	gagcagaguccgacggauctt
	Antisense	gauccgucggacucugcuctt
*Smek2* R 703	Sense	ccaaaagacauagagaautt
	Antisense	auucucuaugucuuuuuggtt
*Smek2* R 2463	Sense	gaacacuugcugagggauutt
	Antisense	aaucccucagcaaguguuctt
Negative control	Sense	auccgcgcgauaguacguatt
	Antisense	uacguacuaucgcgcggautt

Mixture of *Smek2* R 128, *Smek2* R 703, and *Smek2* R 2463 was used for *Smek2* knockdown to enhance siSmek2 effectiveness.

### Determination of hepatic mRNA levels

Complementary DNA for real-time reverse transcription polymerase chain reactions (RT-PCR) was synthesized from total RNA (1.0 μg) using Transcriptor First Strand cDNA Synthesis Kits (Roche Holdings AG., Basel, Switzerland). The expression of 10 genes was measured using real-time RT-PCR with SYBR Premix EX Taq II kits and a Thermal Cycler Dice Real Time System TP800 (Takara, Bio Inc., Kusatsu, Japan). The amplification program comprised 10 s of denaturation at 95 °C, annealing for 15 s, and elongation for 10 s at 72 °C. The mRNA levels were normalized to the internal standard β-glucuronidase gene (*Gusb*). Table 6 shows the primer sequences used for RT-PCR.

**Table 6:** Primers for real-time RT-PCR.

Symbol	Forward	Reverse	Product length (bp)	Annealing temperature (°C)
*Sardh*	GCTGGGTGTAGGTGGAGTTG	CTGGTTGGAGGCGATGAAG	217	62
*Dmgdh*	ACAAGGGCAAGGTGATTGG	CTGGTTCTGGTAGGTTCTGTGAG	193	58
*Bhmt*	TGTGGTAGGGCTCAAATCC	CCATTGTCGGTGTGAACTG	288	60
*Cbs*	CGGTGGTGGATAGGTGGTTC	ACAGAGTCGGGCAGGATGAC	181	60
*Gnmt*	CGCTAAAGAACATCGCAAGC	GTGGGTAGTAAGAGAGCCGAAAC	267	60
*Gamt*	TGAAGAGACCTGGCACACTC	CATCTGAGGGAAGGCATAGTAG	265	60
*Pemt*	TAGCAAGGTGGGAGCAGAG	GCTGGAGAGCACAAACACG	227	58
*Pcyt1a*	TCAAGGAAGAGGAGGAAAGAGG	GGCGTGACCAGAGTGAAATAAG	249	58
*Mthfr*	GAGTGTCTTTGAGGTCTTTGAGC	TGGGAGTTGATGGTGAGGA	175	60
*Smek2*	CTGCATATCAGAAGCAGCAG	ACTGATGGGTCCTTACCTTG	142	62
*Gusb*	GCAGTTGTGTGGGTGAATGGG	GGGGTCAGTGTGTTGTTGATGG	143	62

*Bhmt*, betaine-homocysteine methyltransferase; *Cbs*, cystathionine beta-synthase; *Dmgdh*, dimethylglycine dehydrogenase; *Gamt*, guanidinoacetate methyltransferase; *Gnmt*, glycine N-methyltransferase; *Gusb*, glucuronidase; beta; *Ms*, methionine synthase; *Mthfr*, methylenetetrahydrofolate reductase; *Pcyt*, phosphate cytidylyltransferase; *Pemt*; phosphatidylethanolamine N-methyltransferase; *Sardh*; sarcosine dehydrogenase; *Smek*, suppressor of MEK.

### Determination of serum and hepatic sarcosine levels

Serum and hepatic sarcosine levels were measured using sarcosine assay kits (BioVision, San Francisco, CA, USA). Liver tissues (50 mg) were homogenized in 500 μL of 10 mM Tris–HCl containing 1 mM EDTA (pH 7.4) using a biomasher. Enzymes in the homogenates were deactivated by incubation at 60 °C for 10 min, then serum and hepatic sarcosine levels were measured in 20 and 10 μL of liver homogenates and serum samples, respectively.

### Determination of serum betaine levels

Serum betaine (trimethylglycine) levels were measured using high-performance liquid chromatography (HPLC) as described^42^. Serum samples (50 μL) were mixed with an equal volume of 100 mM KH_2_PO_4_ in screw-top microcentrifuge tubes, then derivatization solution (900 μL; 12.5 mM 18-crown-6, 50 mM 4-bromophenacyl bromide in acetonitrile) was added. The tubes were vacuum-packed in plastic bags and placed in a water bath at 80 °C for 1 h. After cooling to room temperature, the samples were centrifuged at 1000×*g*. Supernatants (15 μL) were directly injected into the HPLC comprising a 600 Controller (Waters Corp., Milford, MA, USA), a 486 Tunable Absorbance Detector (Waters Corp.), Supelcosil™ LC-SCX HPLC column, particle size 5 μm, 25 cm × 4.6 mm) (Sigma-Aldrich, St. Louis, MO, US), mobile phase, acetonitrile:ultra-pure-water (9:1) containing 22 mM choline, at a flow rate of 1.5 mL/min.

#### Determination of serum homocysteine levels

Serum homocysteine levels were measured by using reverse phase HPLC as described^43^. Serum samples (200 μL) were incubated at 4 °C for 30 min in 10% tri-n-butylphosphine in dimethyl formamide (20 μL), then cold trichloroacetic acid (200 μL) was added. Protein was precipitated by centrifugation at 1000×*g* for 5 min. Tubes containing 200 μL of supernatant, 200 μL of 0.25 M boric acid (pH10.5), 100 μL of 1.0 mg/mL SBD-F (fluorescent substance) in 2.5 M boric acid (pH7), were vacuum-packed in plastic bags and shaken at 60 °C for 1 h. Samples were iced and filtered using a Millipore filter (DISMIC-3cp) with 0.45-μm pores (Advantec, Tokyo, Japan). Filtrates (10 μL) were injected into the HPLC (600 Controller; 470 scanning fluorescence detector (excitation and emission, 385 and 515 nm, respectively; Bondasphere 5 μ, C18 300 Å, particle size, 5 μm, 15 cm × 3.9 mm; [all from Waters Corp.], mobile phase, 0.1 M phosphoric acid [pH 3] containing 10% v/v methanol; flow rate, 1 mL/min).

#### Fractionation of hepatic phospholipid levels

Hepatic lipids were extracted as described by Folch et al.^44^. Hepatic phospholipids were fractionated by thin-layer chromatography (TLC) using the solvent described by Wada et al.^45^. Phospholipids separated from hepatic lipids in the following order from the bottom of the TLC plate: lysophosphatidylcholine (LPC), sphingomyelin (SPH), phosphatidylcholine (PC), phosphatidylserine and phosphatidylserine (PS + PI), phosphatidylethanolamine (PE), and neutral lipids (NL). Hepatic lipids (1 μL) were dried, fused to 50 μL of hexane, and spotted onto TLC plates (Merck). The hexane was evaporated from the plates, then the lipids were separated in a chamber containing gas from the solvent (CHCl_3_:MetOH:acetic acid: ultrapure water, 50:30:8:4) until the solvent reached the top of the TLC plates (~ 2 h). The TLC plates were removed from the chamber and dried. Lipids on the plates were visualized by exposing them to iodic gas in another chamber. Thereafter, colored phospholipid bands on the plates were edged with pencil in a fume hood and scraped into test tubes using a razor.

#### Determination of hepatic phospholipid levels

Phospholipids were mixed with 1 mL of 70% perchloric acid in tubes and heated at 180 °C for 30 min to 1 h until the solution became clarified. After cooling to room temperature, the following were added to the tubes in the order: ultrapure water (5 mL), 2.5% ammonium molybdate (1 mL), and 10% ascorbic acid (1 mL). The tubes were vortex-mixed, placed in boiling water for 5 min with a marble as a tap, then cooled to room temperature in a water bath, The absorbance of each solution was measured at 820 nm.

#### Statistical analysis

We extracted and compared genes with altered expression levels in the microarray analysis between ExHC and congenic rats. Values with *P* = 0.01 were considered significantly different. Differences in data of in vivo experiment were assessed using two-way analyses of variance (ANOVA). When interaction between two elements was identified, the data were analyzed using Tukey–Kramer post-hoc tests. Differences in data of in vitro experiment were assessed using Student’s *t*-test. Values with *P* < 0.05 were considered significantly different. Data are shown as means with standard error of the means (SEM). Data were statistically analyzed using Excel 2011 with the Statcel 3 add-in (Microsoft Corp., Redmond, WA, USA).

## Supplementary Information

Supplementary Figure S1.

Supplementary Figure S2.

## Data Availability

The data underlying the results presented in the study are available from Zenodo (https://zenodo.org/) (reserved 10.5281/zenodo.7370257).

## Abbreviations

AGPC: Acid-guanidium-phenol-chloroform
ANOVA: Analyses of variance
BN rat: Brown Norway rat
CREB: CAMP response element-binding protein
CREBBP: CAMP response element-binding protein binding protein
CRTC2: CAMP response element-binding protein-regulated transcription coactivator 2
congenic rat: Congenic ExHC.BN-*Dihc2*^*BN*^ rat
Cy3: Cyanine3
DIHC: Diet-induced hypercholesterolemia
ExHC rat: Exogenously hypercholesterolemic rat
HNF-1a: Hepatocyte nuclear factor-1a
HPLC: High-performance liquid chromatography
LATS1: Large tumor suppressor kinase 1
LPC: Lysophosphatidylcholine
LPDS: Lipoprotein deficient serum
MODY3: Maturity-onset diabetes of the young
NL: Neutral lipids
PAP1: Production of anthocyanin pigment 1
PBS: Phosphate buffered saline
PC: Phosphatidylcholine
PE: Phosphatidylethanolamine
Pfkl: Liver-type phosphofructokinase
PI: Phosphatidylinositol
PS: Phosphatidylserine
RSTS: Rubinstein-Taybi syndrome
SPH: Sphingomyelin
Sardh: Sarcosine dehydrogenase
SD rat: Sprague–Dawley rat
SEM: Standard error of the means
SREBP-1c: Sterol regulatory element binding protein-1c
TLC: Thin-layer chromatography
VLDL: Cholesterol ester-rich very low density lipoprotein
