# Supplementary figures and images for: Mutation in Smek2 regulating hepatic glucose metabolism causes hypersarcosinemia and hyperhomocysteinemia in rats
Source: Sci Rep. 2023 Feb 21;13:3053. doi: 10.1038/s41598-022-26115-z (PMC9944932; doi:10.1038/s41598-022-26115-z)

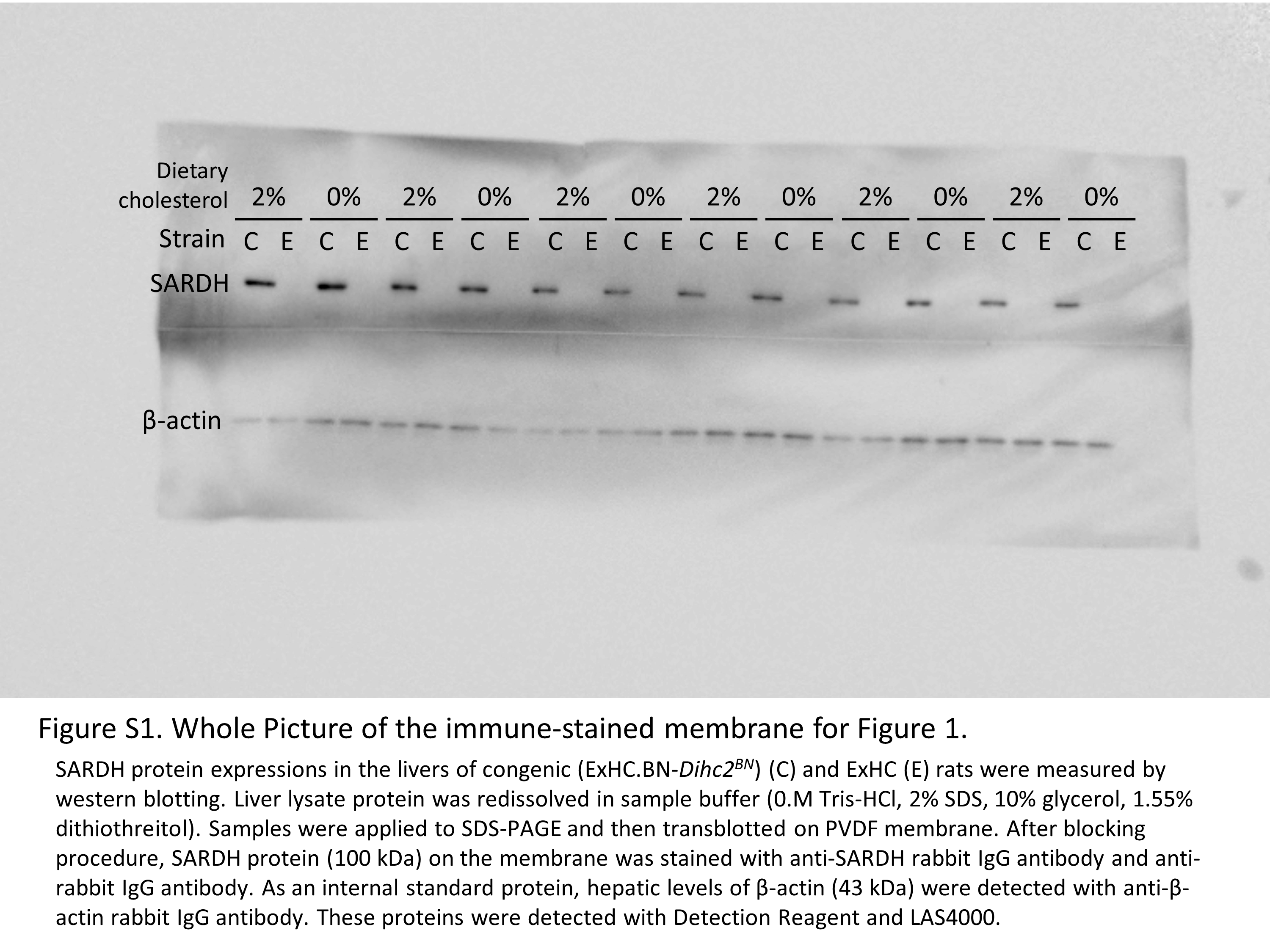

Supplement: Supplementary file 1 — Supplementary Figure S1. [file 41598_2022_26115_MOESM1_ESM.tif]

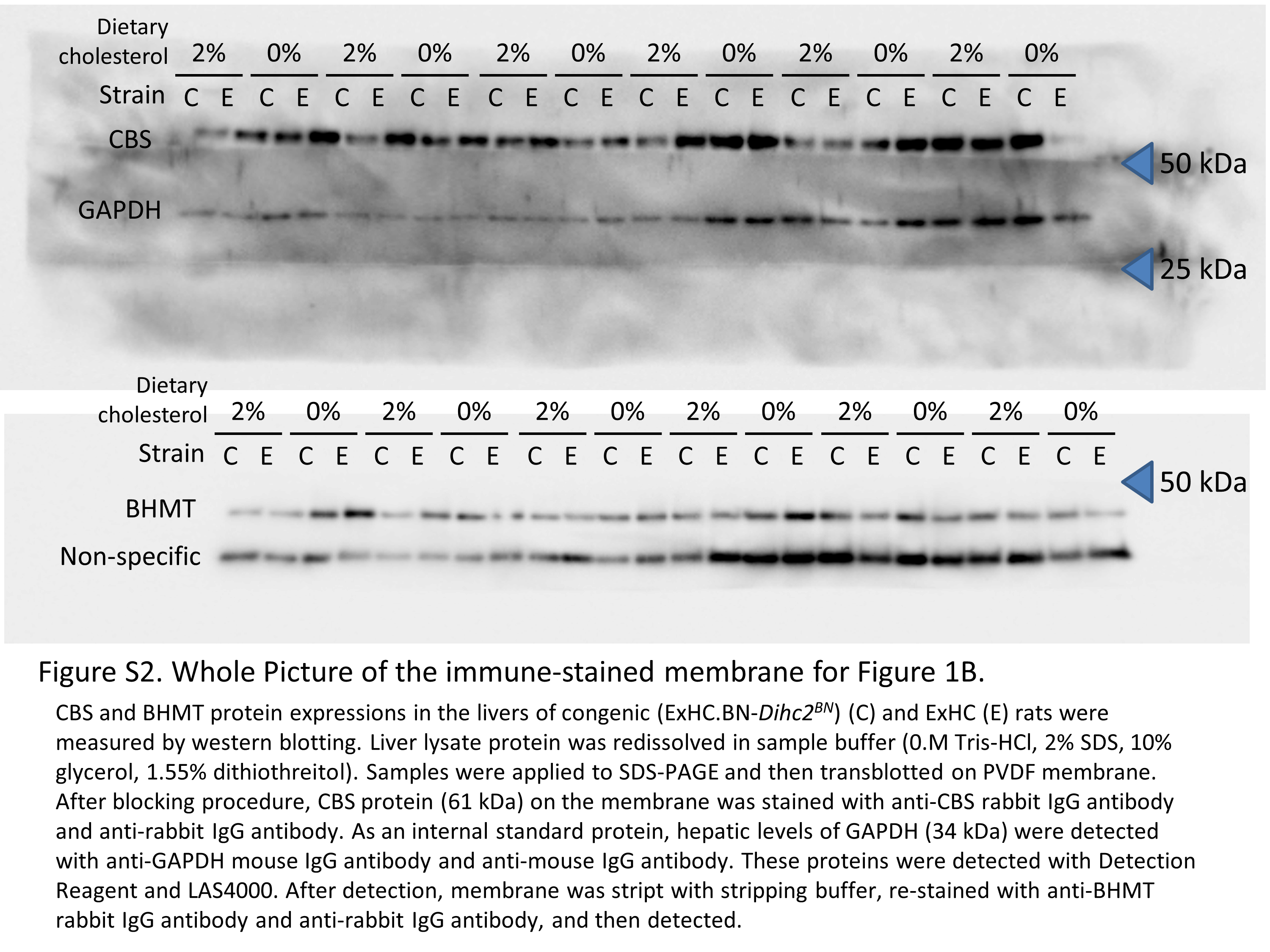

Supplement: Supplementary file 2 — Supplementary Figure S2. [file 41598_2022_26115_MOESM2_ESM.tif]
